# Quantifying uniaxial prestress and waveguide effects on dynamic elastography estimates for a cylindrical rod

**DOI:** 10.1121/10.0022581

**Published:** 2023-12-01

**Authors:** Melika Salehabadi, Lara Nammari, Aime Luna, Joseph Crutison, Dieter Klatt, Thomas J. Royston

**Affiliations:** UIC Richard and Loan Hill Department of Biomedical Engineering, University of Illinois Chicago, 851 South Morgan Street, Chicago, Illinois 60607, USA

## Abstract

Dynamic elastography attempts to reconstruct quantitative maps of the viscoelastic properties of materials by noninvasively measuring mechanical wave motion in them. The target motion is typically transversely-polarized relative to the wave propagation direction, such as bulk shear wave motion. In addition to neglecting waveguide effects caused by small lengths in one dimension or more, many reconstruction strategies also ignore nonzero, non-isotropic static preloads. Significant anisotropic prestress is inherent to the functional role of some biological materials of interest, which also are small in size relative to shear wavelengths in one or more dimensions. A cylindrically shaped polymer structure with isotropic material properties is statically elongated along its axis while its response to circumferentially-, axially-, and radially-polarized vibratory excitation is measured using optical or magnetic resonance elastography. Computational finite element simulations augment and aid in the interpretation of experimental measurements. We examine the interplay between uniaxial prestress and waveguide effects. A coordinate transformation approach previously used to simplify the reconstruction of un-prestressed transversely isotropic material properties based on elastography measurements is adapted with partial success to estimate material viscoelastic properties and prestress conditions without requiring advanced knowledge of either.

## I. INTRODUCTION

Dynamic elastography methods—using noninvasive optical, ultrasonic, or magnetic resonance imaging modalities—aim to quantitatively map the shear viscoelastic properties of materials. These properties in biological tissues are often altered by disease and injury. When considering larger (relative to wavelength) regions of interest, like the liver or brain, boundary effects often can be ignored. However, as elastography expands to other anatomical regions where dimensions in at least one direction are smaller or of comparable length to bulk shear wavelengths—such as in slender skeletal muscles, blood vessels, the heart wall, and the cornea—boundary effects become non-negligible and must be considered. Researchers using optical elastography to assess the viscoelastic properties of the cornea have long recognized this, adapting models to include waveguides by treating the cornea as a plate-like structure. Here, transverse wave motion on the cornea is modeled as Rayleigh-Lamb waves.^1^ Blood vessels, as well, have been modeled using cylindrical shell equations considering fluid-structure interaction.^2–4^ Limited studies on cardiac elastography have also acknowledged the frequency-dependent (i.e., wavelength-dependent) waveguide behavior of the heart wall.^5^

Often, when elastography studies are done under varying nonzero quasi-static prestress conditions, observed changes in mechanical wave behavior are attributed solely to the nonlinear property of the tissue: it is observed that its shear and viscous constants are highly dependent on the static load and associated deformation. A recent article provides a summary of the literature relevant to this issue, in particular for uniaxially prestressed cylindrically-shaped structures, as well as biaxially prestressed plate-like structures.^6^ In another recent article, the impact of neglecting the prestress effect in cornea elastography is quantified.^7^

In the present study, we focus on a uniaxially prestressed cylindrical structure as an idealized geometry for skeletal muscles found throughout the body that have been studied using elastography including, muscles of the lower leg,^8–12^ upper leg,^13–16^ forearm,^17^ and upper arm.^18–20^ We investigate the confounding effects of finite dimensions and prestress on elastography measurements. We articulate and evaluate a strategy for decoupling prestress and waveguide effects from estimates of material shear viscoelastic properties based on circumferentially-, axially-, and transversely-polarized wave motion in the cylinder.

## II. THEORY

### A. Mechanical wave motion in a uniaxially prestressed linear viscoelastic material

Most dynamic elastography methods assume that the measured transverse wave speed or wavelength for small amplitude (linear theory assumption) motion is directly related to the material's elastic or viscoelastic properties.^21^ Assuming isotropy, homogeneity, and neglecting boundary effects or variation in density ρ in a nearly incompressible viscoelastic material, the frequency-dependent shear wave phase speed $c\left[\omega \right]$ for harmonic excitation at circular frequency, ω, is

$$c\left[\omega \right]=\omega /\mathrm{Real}\left[k_{sh}\left[\omega \right]\;\right]=\frac{1/\sqrt{\rho }}{\mathrm{Real}\left[1/\sqrt{\mu \left[\omega \right]}\right]}.$$ (1)

Here, $k_{sh}\left[\omega \right]$ is the complex-valued, frequency-dependent shear wave number, and $\mu \left[\omega \right]$ is the complex-valued, frequency-dependent shear modulus, comprised of the shear storage modulus, $\mu_{R}\left[\omega \right]$, and the shear loss modulus, $\mu_{I}\left[\omega \right]$, such that $\mu \left[\omega \right]=\mu_{R}\left[\omega \right]+j\mu_{I}\left[\omega \right]$ where $j=\sqrt{-1}$. The attenuation rate of the wave as it propagates is governed by the imaginary part of the wavenumber: $\mathrm{Imag}\left[k_{sh}\right]$. In a viscoelastic material, both the shear storage and loss moduli affect both the phase speed and attenuation rate. In a purely elastic material, $\mu_{I}=0$, there is no attenuation and the phase speed is independent of frequency (nondispersive) and reduces to $c=\sqrt{\mu /\rho }$. While some linear studies have assumed pure elasticity (no viscosity), often their analyses are generalizable to the linear viscoelastic problem for harmonic motion by simply adding the imaginary shear loss modulus to form the complex shear modulus. This approach is used in the present analysis.

**FIG. 1.**
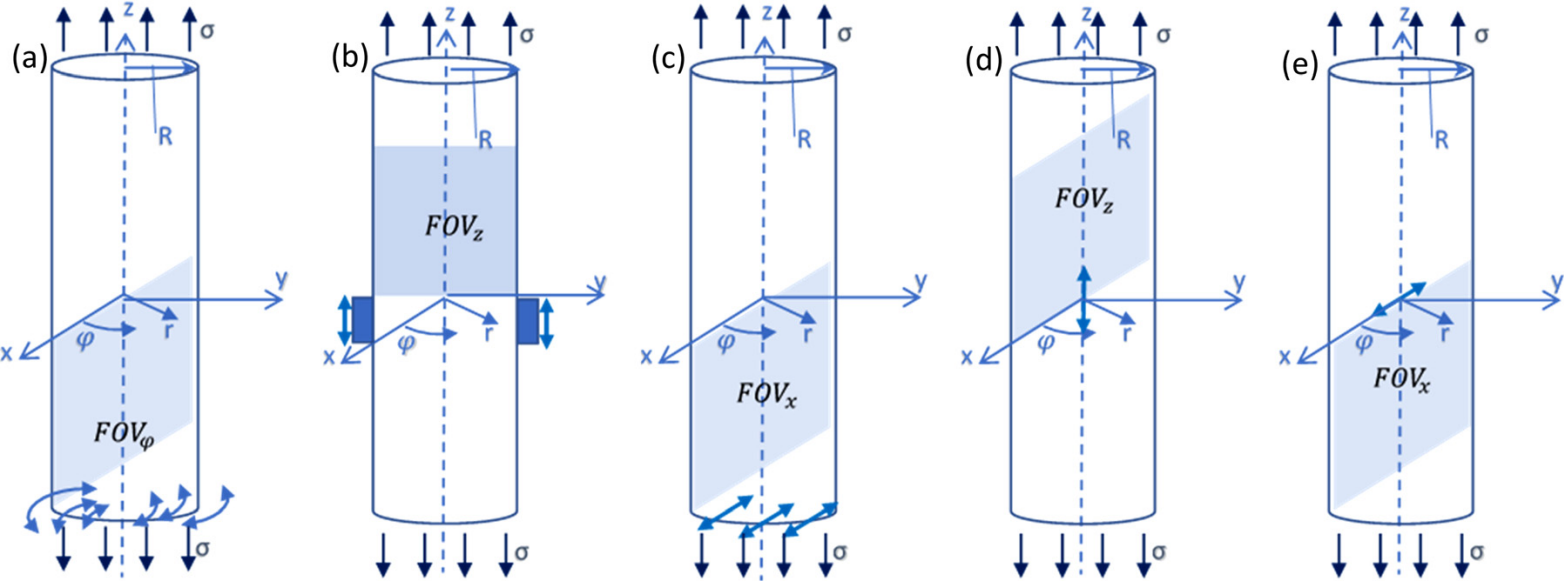
(Color online) Uniaxially prestressed cylinder with different harmonic excitation configurations. Shaded field of view regions ${\mathrm{FOV}}_{\varphi },\;{\mathrm{FOV}}_{z},\;\text{and}\;{\mathrm{FOV}}_{x}$ show the location of images presented in the following figures for torsionally-, axially-, and transversely-polarized wave motion. These are sagittal slices in the *x–z* or *y–z* plane. Deeper shaded blocks with vertical thick blue two-way arrows denote cross section of band surrounding the cylinder that delivers (*z*) axially-polarized harmonic actuation (b). Torsionally-polarized (a) and transversely-polarized (c) motion is input by appropriate motion at the base of the phantom, indicated by arrows. Axially-polarized (d) and transversely-polarized (e) line segment sources are also indicated at the geometric center of the phantom.

Consider the introduction of a uniaxial static prestress σ parallel to the z-axis (Fig. 1). If the static deformation due to the prestress is assumed sufficiently small such that higher order nonlinear terms can be neglected, the governing equations expressed in polar coordinates $\left(r,\varphi ,z\right)$ are as follows: where $\left(u,v,w\right)$ denote incremental displacements in the $\left(r,\varphi ,z\right)$ directions, $S_{rr},\;S_{\varphi \varphi }\;\text{and}\;S_{zz}$ are normal stresses in the respective directions, and $S_{r\varphi },\;S_{\varphi z},\;S_{zr},\;S_{rz},\;S_{z\varphi }\;\text{and}\;S_{\varphi r}$ are shear stresses with the first subscripted dimension referring to the shear surface and the second subscripted dimension denoting the shear direction. Finally, subscripted $\left(r,\varphi ,z,t\right)$ after a comma refer to partial derivatives with respect to that spatial or time dimension,^22^

$$S_{rr,r}+\frac{1}{r}\left(S_{rr}-S_{\varphi \varphi }+S_{\varphi r,\varphi }\right)+S_{zr,z}=\rho u_{,tt},$$ (2)

$$S_{r\varphi ,r}+\frac{1}{r}\left(S_{\varphi r}+S_{r\varphi }+S_{\varphi \varphi ,\varphi }\right)+S_{z\varphi ,z}=\rho v_{,tt},$$ (3)

$$S_{rz,r}+\frac{1}{r}\left(S_{rz}+S_{\varphi z,\varphi }\right)+S_{zz,z}=\rho w_{,tt}.$$ (4)

The normal and shear stress terms are as follows:

$$S_{rr}=\left(\lambda +2\mu \right)u_{,r}+\frac{\lambda }{r}\left(v_{,\varphi }+u\right)+\left(\lambda +\lambda_{\sigma }\sigma \right)w_{,z},$$ (5)

$$S_{\varphi \varphi }=\lambda u_{,r}+\left(\lambda +2\mu \right)\frac{1}{r}\left(v_{,\varphi }+u\right)+\left(\lambda +\lambda_{\sigma }\sigma \right)w_{,z},$$ (6)

$$S_{zz}=\left(\lambda +\lambda_{\sigma }\sigma \right)u_{,r}+\frac{\left(\lambda +\lambda_{\sigma }\sigma \right)}{r}\left(v_{,\varphi }+u\right)+\left(\lambda +2\mu +\sigma +4\mu_{\sigma }\sigma +2\lambda_{\sigma }\sigma \right)w_{,z},$$ (7)

$$S_{r\varphi }=\frac{\mu }{r}\left(u_{,\varphi }-v\right)+\mu v_{,r},$$ (8)

$$S_{\varphi z}=\left(\mu +\mu_{\sigma }\sigma \right)v_{,z}+\left(\mu +\mu_{\sigma }\sigma \right)\frac{1}{r}w_{,\varphi },$$ (9)

$$S_{zr}=\left(\mu +\mu_{\sigma }\sigma \right)w_{,r}+\left(\mu +\sigma +\mu_{\sigma }\sigma \right)u_{,z},$$ (10)

$$S_{rz}=\left(\mu +\mu_{\sigma }\sigma \right)u_{,z}+\left(\mu +\mu_{\sigma }\sigma \right)w_{,r,}$$ (11)

$$S_{z\varphi }=\left(\mu +\mu_{\sigma }\sigma \right)\frac{1}{r}w_{,\varphi }+\left(\mu +\sigma +\mu_{\sigma }\sigma \right)v_{,z},$$ (12)

$$S_{\varphi r}=\mu v_{,r}+\frac{\mu }{r}\left(u_{,\varphi }-v\right).$$ (13)

Here, λ is the volume elasticity of the material, a Lame constant. It is closely related to the material's bulk modulus κ=λ+(2/3)μ. In biological soft tissue or other “nearly incompressible” materials, λ and κ are multiple orders of magnitude greater than μ such that the Poisson's ratio for the material approaches, but does not equal 0.5. The terms $\mu_{\sigma }$ and $\lambda_{\sigma }$ are coefficients that regulate material behavior in the presence of an initial stress, which in this case is uniaxial prestress σ aligned with the *z* (cylinder) axis, as shown in Fig. 1. Specifically, they account for a linear dependence of μ and λ on σ. In the Refs. 22 and 23, these terms are $\beta_{1}$ and $\beta_{2}$, and $\beta_{1}$ is related to *A* used in other references,^24–26^ where its value in soft tissue-like nearly incompressible materials can range between negative and positive values. It has been hypothesized that its value depends on microstructure and thus can reveal material changes not captured by μ.^24^ As noted, the previous formulation neglects higher (3rd, 4th, etc.) order nonlinear terms in the strain energy function and the limitations of this simplification will be evident in the numerical and experimental studies of Secs. III and IV. Incorporation of the higher order terms, which have been detailed in previous studies investigating bulk wave motion without waveguide effects,^23^ is left for future study. Such an incorporation has been successfully detailed in other elastography-focused studies, in particular, to understand the impact of compressive uniaxial loading.^24–26^

In the present study, we will assume harmonic steady state motion. By using the separation of variables one form of the solution for u,v,and w, the symmetric form, is

$$u\left[r,\varphi ,z,t\right]=U\left[r\right]\;\text{cos}\left[n\varphi \right]e^{j\left(\omega t-\xi z\right)},$$ (14)

$$v\left[r,\varphi ,z,t\right]=-V\left[r\right]\;\text{sin}\left[n\varphi \right]e^{j\left(\omega t-\xi z\right)},$$ (15)

$$w\left[r,\varphi ,z,t\right]=W\left[r\right]\;\text{cos}\left[n\varphi \right]e^{j\left(\omega t-\xi z\right)}.$$ (16)

The anti-symmetric form is

$$u\left[r,\varphi ,z,t\right]=U\left[r\right]\;\text{sin}\left[n\varphi \right]e^{j\left(\omega t-\xi z\right)},$$ (17)

$$v\left[r,\varphi ,z,t\right]=V\left[r\right]\;\text{cos}\left[n\varphi \right]e^{j\left(\omega t-\xi z\right)},$$ (18)

$$w\left[r,\varphi ,z,t\right]=W\left[r\right]\;\text{sin}\left[n\varphi \right]e^{j\left(\omega t-\xi z\right)}.$$ (19)

In the following subsections, we consider specific cases of torsionally-, axially-, and transversely-polarized harmonic wave excitation that leads to simplifications of the previous expressions.

### B. Torsionally-polarized wave front

Following Section 8.2.2 of Graff,^27^ consider waves that are torsionally-polarized and axisymmetric, meaning no variation with φ. The anti-symmetric Eqs. (17)–(19) reduce to the case of n=0, and thus $u\left[r,\varphi ,z,t\right]=w\left[r,\varphi ,z,t\right]=0$. Furthermore, we can write

$$v\left[r,z,t\right]=V\left[r\right]e^{j\left(\omega t-\xi z\right)}=\left\{\begin{array}{l} Are^{j\left(\omega t-\xi z\right)},\\ AJ_{1}\left[\beta r\right]e^{j\left(\omega t-\xi z\right)}, \end{array}\right.\;\begin{array}{c} \beta =0,\\ \beta >0, \end{array}$$ (20)

where $J_{1}$ denotes a Bessel function of the first kind of order 1. The radius of the cylinder will determine allowable values of β>0, for which there will be an infinite number such that, in general,

$$v\left[r,z,t\right]=A_{0}re^{j\left(\omega t-\xi z\right)}+\sum_{n=1}^{\infty }A_{n}J_{1}\left[\beta_{n}r\right]e^{j\left(\omega t-\xi_{n}z\right)}.$$ (21)

At =R, the cylinder boundary, $S_{r\theta }=(\mu /r)\left(u_{,\theta }-v\right)+\mu v_{,r}=0$ which means that $v_{,r}-(1/r)v=0$. This is satisfied for the case that β=0 and also imposes that $\beta RJ_{0}\left[\beta R\right]=J_{1}\left[\beta R\right]$, which leads to values $\beta_{1}R=5.136,\;\beta_{2}R=8.417,\beta_{3}R=11.62,\;\ldots$ While there are an infinite number of solution sets, given attenuation due to viscosity we expect the lowest wavenumber values, associated with the longest wavelengths, to dominate as we move further from the source of excitation. Additionally, the geometric configuration of the source of the excitation may preferentially drive certain wavenumber solutions.

With this form for $v\left[r,z,t\right]$, Eq. (3) reduces to the following:

(22)
$$\mu \left(V_{,rr}-\frac{1}{r}V_{,r}\right)+\frac{2\mu }{r}\left(V_{,r}-\frac{1}{r}V\right)-\xi^{2}\left(\mu +\sigma +\mu_{\sigma }\sigma \right)V=-\omega^{2}\rho V,$$ (22a)

$$\beta^{2}+\xi^{2}\left(1+\frac{\sigma }{\mu }\left(1+\mu_{\sigma }\right)\right)=k_{sh}^{2}.$$ (22b)

Here, ξ and β denote complex wavenumbers, with ξ in the *z-*direction, and β in the *r*-direction. Equation (22) shows how they are related to the shear wave number $k_{sh}$. For the case that β=0 and σ=0 we have $\xi_{0}=k_{sh}$. In other words, the torsional waves propagate along the cylinder axis exactly as bulk shear waves, with wavelength $\lambda_{sh}$ equal to $2\pi /\mathrm{Real}\left[k_{sh}\right]$. If there is an axial prestress σ then the wavelength will equal

$$\lambda_{\xi_{0}}=\frac{2\pi }{\mathrm{Real}\left[\xi_{0}\right]}=\frac{2\pi }{\mathrm{Real}\left[k_{sh}\right]}R\text{eal}\left[\sqrt{1+\frac{\sigma }{\mu }\left(1+\mu_{\sigma }\right)}\right].$$ (23)

Assuming the material to be nearly incompressible, axial prestress σ and axial prestrain ϵ are related by $\sigma =3\mu_{0}\epsilon$, where $\mu_{0}$ is the static (real number) value of $\mu \left[\omega \right]$ at ω=0. If one is able to acquire estimates of $\xi_{0}$ based on fitting $e^{-j\xi_{0}z}$ to the measured wave profile along the cylinder axis *z* at a given r and φ at both unstressed and prestressed conditions at multiple frequencies, then $\mu \left[\omega \right]\;\text{and}\;\sigma$ can be independently determined, even without being able to directly measure σ under static load conditions. (A direct measurement of σ may not be possible *in vivo* or in any situation where the cylindrical structure is connected at its ends to another structure and it is impossible to measure the stress condition between them.) Specifically, by selectively driving (or measuring via appropriate filtering) only the β=0 mode, measurements at multiple frequencies without prestress enable a determination of $\mu \left[\omega \right]$, including (possibly by extrapolation with an assumed rheological model) $\mu_{0}=\mu \left[\omega =0\right]$. Then, we can use the fact that $\sigma =3\mu_{0}\epsilon$ and Eq. (23) to independently determine values for $\sigma \;\text{and}\;\mu_{\sigma }$, assuming we can estimate ϵ from imaging. Numerical and experimental studies in the Secs. III and IV, respectively, illustrate and evaluate this process.

### C. Axially-polarized axisymmetric wave front

We next consider the case of an axisymmetric axially-polarized wave front, which theoretically could be driven by axial oscillation of the rigid cuff encircling the cylinder over a finite axial length. This again results in *n* = 0, but now with the symmetric form, Eqs. (14)–(16), which reduce to

$$u\left[r,z,t\right]=U\left[r\right]e^{j\left(\omega t-\xi z\right)},$$ (24)

$$w\left[r,z,t\right]=W\left[r\right]e^{j\left(\omega t-\xi z\right)}.$$ (25)

Following Graff,^27^ Section 8.2.2, the boundary condition at the outer radius *R* means that $S_{rz}=S_{rz}=0$, which is satisfied by the following forms for $U\left[r\right]$ and $W\left[r\right]$:

$$U\left[r\right]=\left(j\xi J_{1}\left[\beta r\right]+A\alpha J_{1}\left[\alpha r\right]\right),$$ (26)

$$W\left[r\right]=\left(\beta J_{0}\left[\beta r\right]+Aj\xi J_{0}\left[\alpha r\right]\right).$$ (27)

Here again, there will be certain allowable values of $\alpha_{1},\;\alpha_{2},\ldots ,\;\alpha_{\infty }$, $\beta_{1},\;\beta_{2},\ldots ,\;\beta_{\infty }$, and $\xi_{1},\;\xi_{2},\ldots ,\;\xi_{\infty }$. Inserting these forms for u and w into Eq. (4) results in the following, where Bessel functions with α and β are separated into two separate equations, Eqs. (28) and (29), that both must be satisfied,

$$\left(1+\mu_{\sigma }\frac{\sigma }{\mu }\right)\beta^{2}+\left(1+\frac{\sigma }{\mu }\left(1+3\mu_{\sigma }+\lambda_{\sigma }\right)\right)\xi^{2}=\frac{\omega^{2}\rho }{\mu }=k_{sh}^{2},$$ (28)

$$\left(1+\frac{\sigma }{\lambda +2\mu }\left(\lambda_{\sigma }+2\mu_{\sigma }\right)\right)\alpha^{2}+\left(1+\frac{\sigma }{\lambda +2\mu }\left(1+4\mu_{\sigma }+2\lambda_{\sigma }\right)\right)\xi^{2}=\frac{\omega^{2}\rho }{\lambda +2\mu }=k_{p}^{2}.$$ (29)

Equation (28) shows the coupled relationship between axial wavenumber ξ, radial wavenumber β, and shear wavenumber $k_{sh}$. Equation (29) relates ξ, another axial wavenumber α, and compression (longitudinal) wavenumber $k_{p}$. If prestress σ=0, the previous equations reduce to expressions found in Graff.^27^

Unlike in the torsional case, even with selective excitation of, say, the lowest values for $\alpha_{1}$, $\beta_{1},$ and $\xi_{1}$, there is not a case with α or β=0. Nonetheless, Eqs. (28) and (29) do show the relationship between shear, compression, axial, and radial wavenumbers, and how this is effected by uniaxial prestress. Focusing on Eq. (28), if values for $\beta_{1}$ and $\xi_{1}$ can be determined at multiple frequencies with no prestress present, they can be used to then estimate $\mu \left[\omega \right]$. Then, prestress is added to determine $\sigma =3\mu_{0}\epsilon$ based on measuring ϵ. Following that, Eq. (28) can be used at different prestress levels to estimate $\mu_{\sigma }$ and $\lambda_{\sigma }$. Numerical and experimental studies in the following Secs. III and IV, respectively, illustrate and evaluate this process.

### D. Transversely-polarized axisymmetric wave front

For wave symmetric to the *x*-axis in Fig. 1, we restrict ourselves to the case of *n* = 1, and Eqs. (14)–(16) reduce to the following:

$$u\left[r,\varphi ,z,t\right]=U\left[r\right]\;\text{cos}\left[\varphi \right]e^{j\left(\omega t-\xi z\right)},$$ (30)

$$v\left[r,\varphi ,z,t\right]=-V\left[r\right]\;\text{sin}\left[\varphi \right]e^{j\left(\omega t-\xi z\right)},$$ (31)

$$w\left[r,\varphi ,z,t\right]=W\left[r\right]\;\text{cos}\left[\varphi \right]e^{j\left(\omega t-\xi z\right)}.$$ (32)

Unfortunately, this case does not simplify to the same degree as did the torsionally- and axially-polarized cases. All three displacements *u*, *v*, and *w*, are present, and the resulting forms for $U\left[r\right]$, $V\left[r\right]$, and $W\left[r\right]$ remain more complex.^27^ Further analytical development of these solution forms in the presence of uniaxial prestress is left for future study. In the present article, we instead consider simplifying theories that may yield insight as the cylinder's radius becomes small as compared to the wavelengths being considered. We specifically review “thin” and “thick” beam theories under uniaxial prestress conditions.

#### 1. Prestressed one-dimensional “thin” waveguide under transverse excitation

By assuming that the radius *R* of the cylinder is small enough such that βR≪1, then so-called one-dimensional beam theory approximations can be applied. The simplest of these is the Euler–Bernoulli thin beam theory, which allows for the incorporation of prestress σ. Referring again to Fig. 1, the pre-tensioned Euler-Bernoulli thin beam theory described in Section 3.3.4 of Graff^27^ for *x*-polarized transverse wave propagation of the beam along its *z*-axis is the following:

$$EIu_{,\text{zzzz}}-\sigma Au_{,zz}+A\rho u_{,tt}=0.$$ (33)

Here, I is the area moment of inertia about the *y*-axis $[I=(\pi /4)R^{4}$ for a circular cross section of radius *R*], and A is the cross-sectional area in the *x–y* plane. The general solution form is: $v=Ve^{j(\omega t-\xi z)}$ where ξ has four possible solutions,

$$\xi =\begin{array}{c} +\\ - \end{array}\alpha ,\;\begin{array}{c} +\\ - \end{array}j\beta ,\;\alpha ={\left\{-\gamma +{\left(\gamma^{2}+\frac{\omega^{2}}{a^{2}}\right)}^{1/2}\right\}}^{1/2},\beta ={\left\{\gamma +{\left(\gamma^{2}+\frac{\omega^{2}}{a^{2}}\right)}^{1/2}\right\}}^{1/2},\;\gamma =\frac{32}{3R^{2}}\frac{\sigma }{\mu },a=\frac{R}{8}\sqrt{\frac{3\mu }{\rho }}.$$ (34)

We have two propagating waves (*α*) in the + or –*z*-direction, and two non-propagating (near field or evanescent) waves (*β*) in the + or –*z* direction. For the propagating waves, the phase speed will be: $c_{ph}=(\omega /\mathrm{Real}\left[\alpha \right])$. Taking the limit that σ/μ $\ll 3R^{2}/32$ we see that $\alpha ={\left(\omega /a\right)}^{1/2},$ and thus for the elastic case, $c_{ph}=(\omega^{1/2}/2)(3\mu R^{2}/{4\rho )}^{1/4}$, which is the classic thin (Euler–Bernoulli) beam transverse vibration solution. On the other hand, taking the limit of tension σ/μ $\gg 3R^{2}/32$ we then drop $EIu_{,\text{zzzz}}$ from Eq. (33) and reformulate the solution to find that there are two propagating solutions with $c_{ph}={\left(\sigma /\rho \right)}^{1/2}$. This is the classic transverse thin string vibration solution. A case between the extremes of either neglecting σA or EI still does not match the phase speed of bulk shear waves, given by Eq. (1).

#### 2. Prestressed one-dimensional “thick” waveguide under transverse excitation

The thin beam formulation presented in Sec. II D 1 is only a reasonable approximation when the wavelength of the propagating transverse waves is at least an order of magnitude greater than the beam's cross-sectional radius *R*, or equivalent radius for a noncircular cross section. A formulation yielding a valid approximation for shorter transverse waves is based on the Timoshenko beam theory, which allows for shear deformation and accounts for rotational inertia. Incorporating prestress into the formulation for Timoshenko beam theory given in Section 3.4 of Graff,^27^ we have the following:

$$EIu_{,\text{zzzz}}-\sigma Au_{,zz}+A\rho u_{,tt}-I\rho \left(1+\frac{E}{\chi \mu }\right)u_{,\text{zztt}}+\frac{\rho^{2}I}{\chi \mu }u_{,\text{tttt}}=0.$$ (35)

Here, χ is the Timoshenko shear coefficient, equal to 10/9 for a circular cross section. Consider harmonic motion at frequency ω and the general solution form is $u=Ue^{j(\omega t-\xi x)}$, and ξ has four possible solutions,

$$\begin{array}{c} \xi^{4}EI+\xi^{2}\left(\sigma A-\omega^{2}I\rho \left(1+\frac{E}{\kappa \mu }\right)\right)-\omega^{2}\left(\rho A-\omega^{2}\frac{\rho^{2}I}{\kappa \mu }\right)=0,\\ \xi =\begin{array}{c} +\\ - \end{array}{\left\{-\gamma \begin{array}{c} +\\ - \end{array}{\left(\gamma^{2}+\frac{\omega^{2}}{a^{2}}\right)}^{1/2}\right\}}^{1/2},\;\gamma =\frac{8\mu_{0}}{D^{2}\mu_{f}}\epsilon_{L}-\omega^{2}\frac{37\rho }{60\mu },\;a=\sqrt{\frac{3\mu /\rho }{\frac{16}{D^{2}}-\omega^{2}\frac{9\rho }{10\mu }}} \end{array}.$$ (36)

In this case, we cannot separate the wavenumber solutions into propagating and non-propagating pairs. Rather, there will be certain prestress-dependent frequency ranges where we have only one propagating pair and others where we have two propagating pairs.

### E. Accounting for prestress using “transformation acousto-elastography” (TAE)

Equations (23), (28), and (29) “hint” at an alternative strategy to solve the inverse problem of independently identifying inherent material shear viscoelastic properties and prestress based on measurements of wave motion. In previous studies on *transversely isotropic* materials not under prestress,^28–32^ the last author has shown that, by distorting the geometry based on direction and polarization-dependent planar phase speeds one can then solve an equivalent isotropic problem. This approach to the anisotropic problem is “Transformation Elastography.” Uniaxial prestress causes a similar, though not identical, direction and polarization dependence of the planar shear wave phase speed. The same approach is adapted to the acoustoelastic problem here, which also has the added complexity of waveguide effects, and is evaluated in numerical and experimental studies noted in the following.

The analysis in the previous sections shows that the case of torsionally-polarized waves may be the simplest and is a good place to start. Considering that the material is nearly incompressible, the prestress results in a static strain of the cylindrical phantom of unstressed length L and radius R, changing its axial length to $L\left(1+\epsilon \right)=L(1+(\sigma /3\mu_{0}))$ and its radius to $R/\sqrt{1+\epsilon }=R/\sqrt{1+(\sigma /3\mu_{0})}$. By driving or measuring only β=0 torsional wave motion, if one then distorts the axial length by dividing it by $\mathrm{Real}\left[\sqrt{1+(\sigma /\mu )\left(1+\mu_{\sigma }\right)}\right]=\mathrm{Real}\left[\sqrt{1+(3\mu_{0}\epsilon /\mu [\omega ])\left(1+\mu_{\sigma }\right)}\right]$, then fits a solution to $e^{-j\xi_{0}^{*}z}$ in order to identify $\xi_{0}^{*}$, where an asterisk * is added to denote this is based on a distorted geometry, then we see that $\xi_{0}^{*}=k_{sh}$, where $k_{sh}$ was obtained under the unstressed condition. From the degree of distortion needed to achieve this equality, we directly determine $\mu_{\sigma }$.

Note, $\mu \left[\omega \right]$ ( $\mu_{0},\;\mu_{\alpha },\;\alpha$) needs to be determined for the no prestrain ( ϵ=0) case for multiple frequencies. So, instead of adjusting the geometry distortion by varying $\mu_{\sigma }$ as we propose here to match with the unstressed case, another approach is to adjust $\mu_{\sigma }$ to match the measured $\xi_{0}$ in the undistorted geometry using Eq. (23). This will lead to the same result as arrived at using the geometry distortion (TAE approach). However, we propose that the TAE concept, applicable to more than the torsional polarization case, may help conceptually as an analysis tool.

Next, consider the axisymmetric, axially-polarized wavefront. In this case, the geometry is distorted by dividing the axial length by $\text{Real}\left[\sqrt{1+(3\mu_{0}\varepsilon /\mu [\omega ])(1+3\mu_{\sigma }+\lambda_{\sigma })}\right]$ and dividing the diameter or radius by $\text{Real}\left[\sqrt{1+\mu_{\sigma }(3\mu_{0}\varepsilon /\mu [\omega ])}\right]$, such that Eq. (28) simplifies to

$$\beta^{*2}+\xi^{*2}=k_{sh}^{2}.$$ (37)

Assume that, within the region bounded by the rigid cuff, we have purely radially converging axially-polarized waves that can be fit to $J_{0}\left[\beta^{*}r\right]$ in order to solve for $\beta^{*}$. This follows from Eq. (27) since it is assumed that $\xi^{*}=0$. Additionally, with this assumption, we see from Eq. (37) that $\beta^{*}=k_{sh}.$ Thus, like in the torsionally-polarized case, we can match $\beta^{*}$ to the value of $k_{sh}$ that was obtained with no prestress by adjusting $\mu_{\sigma }$. This becomes another means of identifying $\mu_{\sigma }$.

Then, by measurement of the wave field generated in both the radial and axial directions with and without axial prestress present, we can use Eqs. (26)–(29) to estimate $\lambda_{\sigma }$. However, there are an infinite number of solutions to this transcendental set of equations. Numerical and experimental studies in Sections III and IV evaluate whether this approach can be used to determine $\lambda_{\sigma }$. Furthermore, while a similar analysis has not been completed for the case of transversely-polarized (flexural) waves, numerical studies detailed in the following provide insight into the relationship between prestress and $\mu_{\sigma }$ and $\lambda_{\sigma }$ in this case.

## III. NUMERICAL CASE STUDY

### A. Methods

An analytical and numerical case study was conducted to understand the interactions between uniaxial prestress and waveguide behavior, as well as to evaluate the TAE approach introduced in Sec. II E. Wave motion in the cylindrical phantom shown in Fig. 1, with parameter values provided in Table I, was simulated with a numerical finite element (FE) approach using ANSYS Mechanical APDL Version 2022 R1 (Ansys, Canonsburg, PA). These parameter values were chosen to be similar (though not exactly matching) to those in experimental studies described in Sec. IV. For simulation of torsionally- and axially-polarized harmonic excitation, an axisymmetric mixed u-P formulation was used with Plane183 8-node elements with individual element side dimensions of 0.5 mm. (Both displacements and hydrostatic pressure are taken as primary unknowns in the mixed u-P formulation, which is recommended for nearly incompressible materials.) For transverse *x*-polarized excitation [referring to Fig. 1(c)] a mixed u-P formulation was used with Solid273 8-node by six circumferential plane generalized axisymmetric elements with individual element side dimensions in the axial and radial direction of 0.5 mm.

**TABLE I.** Parameter values for case studies.

Parameter	Symbol	Value(s)
Bulk Modulus	κ	2 GPa
Static shear storage modulus	$\mu_{0}$	27 kPa
Spring pot parameter values	$\mu_{\alpha }$	$1.5\;\mathrm{kPa}\cdot s^{\alpha }$
	α	0.3
Undeformed phantom length	L	100 mm
Undeformed phantom radius	R	10, 17.5 mm
Uniaxial tensile strain	$\epsilon_{L}$	0, 0.025, 0.05, 0.1, 0.2
Density	ρ	1070 $\mathrm{kg}/m^{3}$
Gent model limiting parameter	$J_{m}$	50
Best fit prestress coefficient (FEM)	$\mu_{\sigma }$	−0.75
Best fit prestress coefficient (FEM)	$\lambda_{\sigma }$	2.00

In all cases, first, the response to the static preload is solved by accounting for geometric nonlinearity (nlgeom,on). One end of the cylinder was fixed in the axial (*z*)-direction and the other end was incrementally displaced in the *z*-direction, solving the problem in steps until the desired end displacement was reached that resulted in a uniform uniaxial strain throughout the model. This was done using the Gent model^33^ defined by, $\kappa ,\;\mu_{0},\;\text{and}\;J_{m}$, or using linear elastic properties, $E,\;\nu ,\;\text{and}\;\mu_{0}$, where Young's modulus E and Poisson's ratio ν were chosen to be consistent with the value of κ used for the Gent model. For axial strains up to 20%, differences between the Gent and linear model, as quantified by different predicted axial stress values, were about half the percentage of the strain. Specifically, the axial stress predicted by the Gent model exceeded the stress predicted by the linear model by 1.25% for 2.5% axial prestrain, 2.54% for 5% prestrain, and 11.2% for 20% prestrain. While the Gent model is more accurate, it was necessary to use the linear model in Ansys for the type of linear harmonic perturbation analysis conducted here, which required that the shear modulus value be changed between the static and harmonic analysis phases. This was needed due to the frequency-dependence of the shear modulus.

Specifically, once the static analysis was done, the solution routine was exited and then re-entered, recovering the prestrained geometry and a “total” stiffness matrix that is the sum of the initial stiffness matrix due to material properties plus terms added due to the prestress condition.^34^ Then, the shear and Young's modulus values are updated, which in turn updates the “total” stiffness matrix. This is done before turning geometric nonlinearity off (nlgeom,off) and entering the harmonic solution routine using this modified total stiffness matrix acquired at the end of the static solution routine. Now, harmonic φ, z, or x-direction displacements were applied. For torsional and transverse (flexural) excitation, one end of the phantom was specified to have φ-polarized or x-polarixed motion, respectively, in order to preferentially drive the β=0 torsional mode or n=1 flexural mode, respectively. For axially polarized excitation, nodes on the outer surface of the phantom representing a rigid axially-oscillating cuff in contact with the phantom from 35 to 50 mm axially were given harmonic *z*-polarized displacement inputs. In addition to these simulations, we also computed the response to an axially- and to a transversely-polarized 10 mm long line segment displacement located at the geometric center of the cylinder, as an approximation of dynamic elastography using modulated radiation force of ultrasound to create a force vector at the focal region of the ultrasound probe.

For the harmonic analysis, a “Fractional Voigt” rheological model is assumed^35^ and the shear modulus is modified from $\mu_{0}$ to $\mu \left[\omega \right]=\mu_{0}+\mu_{\alpha }\omega^{\alpha }\;\text{cos}\left[\pi \alpha /2\right]$ and viscous (beta) damping is specified by taking the ratio of the imaginary to the real part of the shear modulus and dividing by ω, the harmonic circular driving frequency. Specifically, the beta damping value is

$$\text{beta}=\frac{\left(\mu_{\alpha }\omega^{\alpha }\;\text{sin}\left[\pi \alpha /2\right]\right)}{\omega \left(\mu_{0}+\mu_{\alpha }\omega^{\alpha }\;\text{cos}\left[\pi \alpha /2\right]\right)}.$$ (38)

### B. Results

**FIG. 2.**
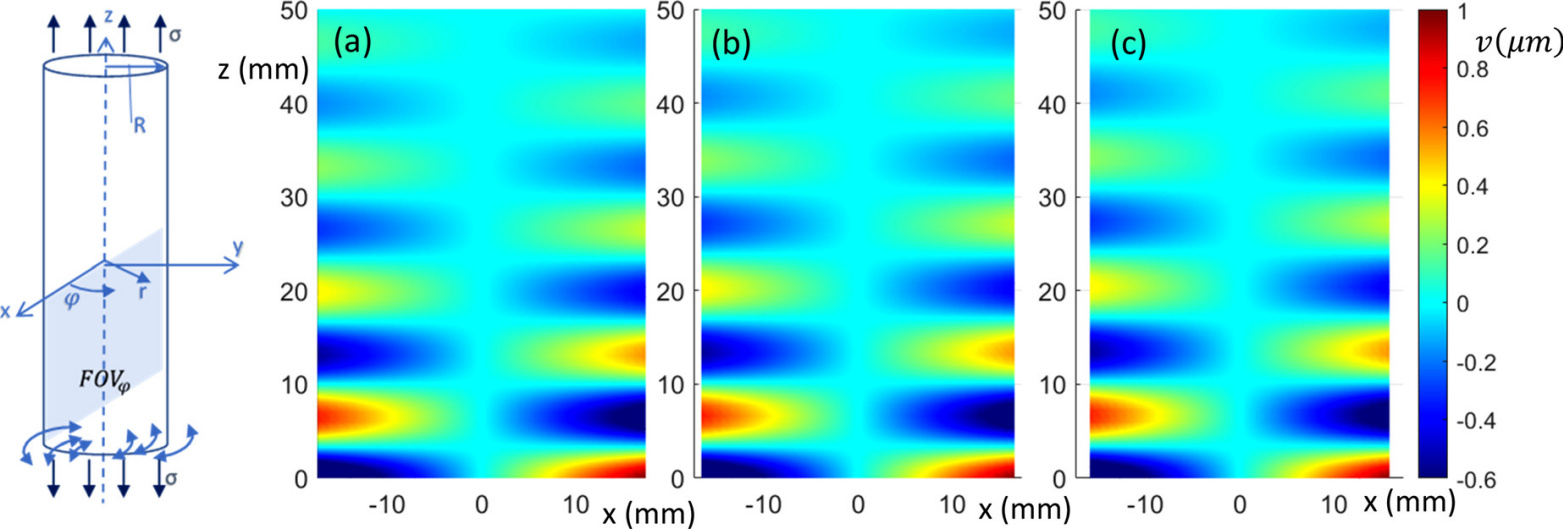
(Color online) Sagittal view of torsionally ( φ)-polarized in-phase wave motion from FE simulation. (a)–(c) 0%, 10%, and 20% axial prestrain, respectively.

**FIG. 3.**
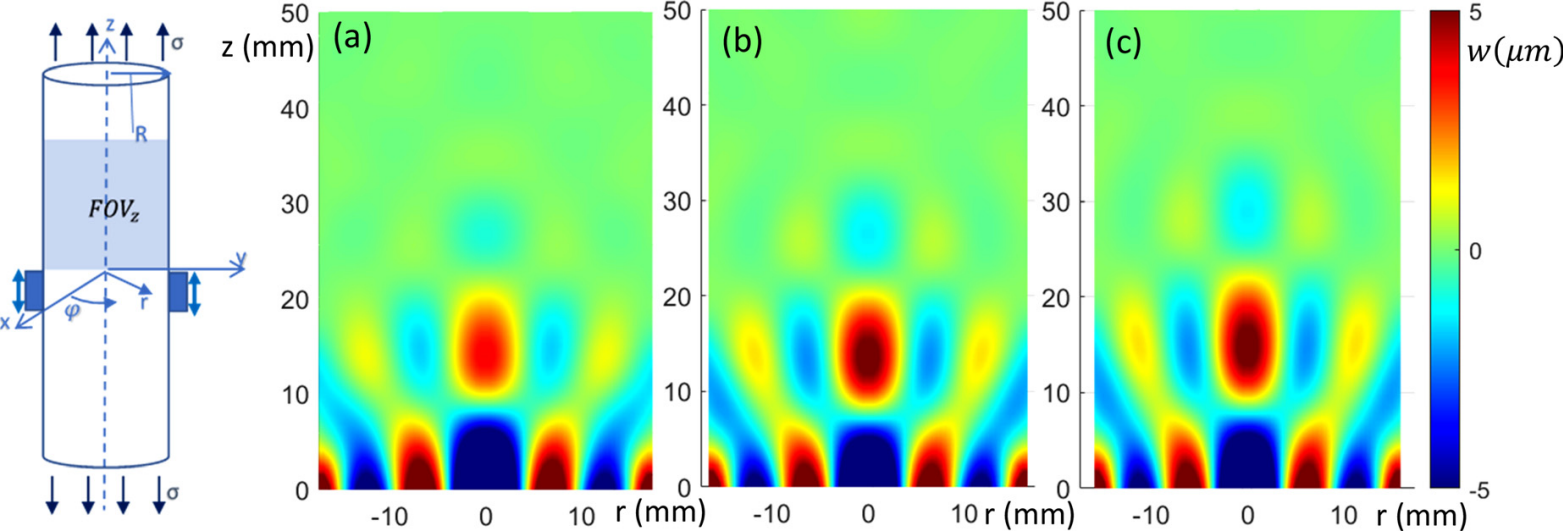
(Color online) Sagittal view of axially (*z*)-polarized in-phase wave motion from FE simulation. (a)–(c) 0%, 10%, and 20% axial prestrain, respectively.

**FIG. 4.**
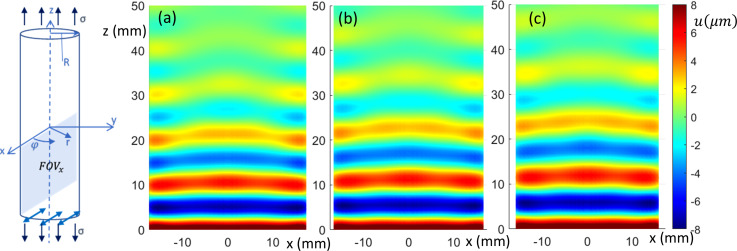
(Color online) Sagittal view of transversely (*x*)-polarized in-phase wave motion from FE simulation. (a)–(c) 0%, 10%, and 20% axial prestrain, respectively.

The FEA-calculated in-phase steady-state torsionally ( φ)-, axially (*z*)-, and transversely (*x*)-polarized response over a sagittal slice (FOVs in Fig. 1) is shown in Figs. 2, 3, and 4, respectively, at 0%, 10%, and 20% axial strain levels at 600 Hz for the 35 mm diameter phantom.

The acoustoelastography challenge is to estimate the complex frequency-dependent shear modulus *and* the prestress and prestress-dependent coefficients, $\mu_{\sigma }\;\text{and}\;\lambda_{\sigma }$, based only on assuming a known density ρ and using the complex wave images at multiple frequencies and prestress levels, from which we can also determine axial prestrain ϵ. The procedures described in Sec. II E are used to estimate the rheological properties first at multiple frequencies under zero prestrain in order to obtain estimates of $\mu_{0},\;\mu_{\alpha },\;\text{and}\;\alpha$, and then with prestrain, in order to estimate prestress σ, and coefficients $\mu_{\sigma }\;\text{and}\;\lambda_{\sigma }$. We start with a curve fit estimate of the wave profile along the *z-*direction during torsionally-polarized harmonic excitation, followed by fits of the wave profile along r- and *z*-directions during axially-polarized harmonic excitation. Transversely-polarized (*x*-direction) and line segment force studies following this are used to identify the effect of prestress σ on flexural wave motion, and to assess the combined prestress and waveguide effect on shear wave motion generated from a focused source, as an approximation of a modulated radiation force of ultrasound source. Results at 600 Hz are summarized in Tables II and III for the 35 and 20 mm diameter phantoms, respectively.

**TABLE II.** Wavenumber estimates for 35 mm phantom at 600 Hz at indicated prestrain levels.

Prestrain $\epsilon_{L}\%$	0	2.5	5	10	20
True Shear modulus, wavenumber	$\mu \left[\omega \right]=\mu_{R}+j\mu_{I}\;\left(\mathrm{kPa}\right)=42.8+j8.05,\;k_{sh}\;\left(m^{-1}\right)=588-j54.9$				
$\xi_{0}$(m^−1^) – torsional (FEM)	588-j54.9	584-j54.6	582-j54.3	577-j53.8	571-j53.3
$k_{sh}=\xi_{0}^{*}$ (m^−1^) – torsional (FEM)	588-j54.9	588-j55.0	588-j54.9	590–j55.0	596-j55.6
β (m^−1^) – axial (FEM)	584-j60.5	596-j62.0	607-j63.5	632-j66.8	686-j74.3
$k_{sh}=\beta^{*}$(m^−1^) – axial (FEM)	584-j60.5	586-j60.9	586-j61.3	588-j62.1	587-j63.6
$\sqrt{\xi^{2}+\beta^{2}}$ (m^−1^) – axial (FEM)	614-j56.4	615-j60.7	618-j64.6	613-j70.4	630-j74.2
$\sqrt{\xi^{*2}+\beta^{*2}}$(m^−1^) – axial (FEM)	614-j56.4	613-j60.5	614-j64.2	599-j68.8	586-j69.0
$\xi_{1}$(m^−1^) – flexural (FEM)	603-j68.6	594–j66.9	585-j65.2	568-j62.1	539-j56.8
$\xi_{1}^{*}$ (m^−1^) – flexural (FEM)	603-j68.6	604-j68.0	604-j67.3	605-j64.1	607-j64.0
$\xi_{1}$(m^−1^) – flexural (“Thin” Theory)	197-j9.18	197-j9.14	197-j9.11	196-j9.04	195-j8.89
$\xi_{1}$ (m^−1^) – flexural (“Thick” Theory)	565-j51.5	564-j51.5	564-j51.4	564-j51.4	563-j51.3
ξ(m^−1^) – trans line source (FEM)	575-j179	569-j177	564-j173	552-j161	526-j136
$k_{sh}=\xi_{a}^{*}$(m^−1^) (FEM)	575-j179	579-j180	582-j179	564-j165	548-j142
$k_{sh}=\xi_{b}^{*}$(m^−1^) (FEM)	575-j179	585-j182	594-j182	587-j171	590-j153
β(m^−1^) – axial line source (FEM)	615-j474	627-j468	641-j464	671-j458	743-j474
$k_{sh}=\beta^{*}$(m^−1^) (FEM)	615-j474	618-j461	622-j450	629-j429	648-j413
$\xi_{0}$(m^−1^) – torsional (Experiment)	583-j54.4	569-j40.1	555-j40.2	540-j52.2	493-j54.4
$k_{sh}=\xi_{0}^{*}$ (m^−1^) $\mu_{\sigma }=-0.5$	583-j54.4	575-j40.5	568-j41.1	565-j54.6	541-j69.7
β (m^−1^) – axial (Experiment)	572-j84.3	NA	586-j38.7	603-j86.0	NA
$k_{sh}=\beta^{*}$(m^−1^) (Experiment)	572-j84.3	NA	573-j37.8	576-j82.1	NA

**TABLE III.** Wavenumber estimates for 20 mm phantom at 600 Hz at indicated prestrain levels.

Prestrain $\epsilon_{L}\%$	0	2.5	5	10	20
Shear modulus & wavenumber	$\mu \left[\omega \right]=\mu_{R}+j\mu_{I}\;\left(\mathrm{kPa}\right)=42.8+j8.05,\;k_{sh}\;\left(m^{-1}\right)=588-j54.9$				
$\xi_{0}$(m^−1^) – torsional (FEM)	588-j54.8	585-j54.5	582-j54.2	577–j53.8	571-j53.2
$k_{sh}=\xi_{0}^{*}$ (m^−1^) – torsional(FEM)	588-j54.8	588-j54.8	588-j54.8	590-j55.0	596-j55.5
β (m^−1^) – axial (FEM)	586-j60.6	598-j61.8	610-j62.9	636-j65.4	694-j73.6
$k_{sh}=\beta^{*}$(m^−1^) – axial (FEM)	586-j60.6	588-j58.7	589-j56.6	592-j51.9	593-j41.8
$\sqrt{\xi^{2}+\beta^{2}}$ (m^−1^) – axial (FEM)	546-j37.5	542-j34.3	536-j25.9	557-j33.8	558-j15.6
$\sqrt{\xi^{*2}+\beta^{*2}}$(m^−1^) – axial (FEM)	546-j37.5	546-j36.8	540-j32.5	567-j41.0	578-j40.7
$\xi_{1}$(m^−1^) – flexural (FEM)	554-j90.4	543-j80.6	534-j72.1	519-j58.3	498-j33.8
$\xi_{1}^{*}$ (m^−1^) – flexural (FEM)	554-j90.4	552-j83.7	551-j77.9	553-j58.4	562-j49.5
ξ (m^−1^) – flexural (“Thin” Theory)	261-j12.1	260-j12.1	260-j12.0	259-j11.8	256-j11.5
ξ (m^−1^) – flexural (“Thick” Theory)	577-j50.7	576-j50.6	575-j50.5	574-j50.3	571-j50.0
ξ(m^−1^) – trans line source (FEM)	552-j123	536-j116	520-j109	492-j98.4	448-j83.4
$k_{sh}=\xi_{a}^{*}$(m^−1^) (FEM)	552-j123	539-j117	526-j111	503-j103	467-j90.9
$k_{sh}=\xi_{b}^{*}$(m^−1^) (FEM)	522-j123	545-j119	537-j116	524-j111	504-j104
β(m^−1^) – axial line source (FEM)	621-j933	627-j937	632-j939	642-j938	660-j888
$k_{sh}=\beta^{*}$(m^−1^) (FEM)	621-j933	619-j919	617-j902	609-j863	590-j737
$\xi_{0}$(m^−1^) – torsional (Experiment)	568-j110	513-j189	499-j94.5	476-j113	484-j59.6
$k_{sh}=\xi_{0}^{*}$ (m^−1^) $\mu_{\sigma }=-0.5$	568-j110	518-j194	509-j101	495-j126	525-j81.6
β (m^−1^) – axial (Experiment)	574-j73.5	NA	606-j80.2	611-j88.2	NA
$k_{sh}=\beta^{*}$(m^−1^) (Experiment)	574-j73.5	NA	593-j72.5	584-j72.0	NA

**FIG. 5.**
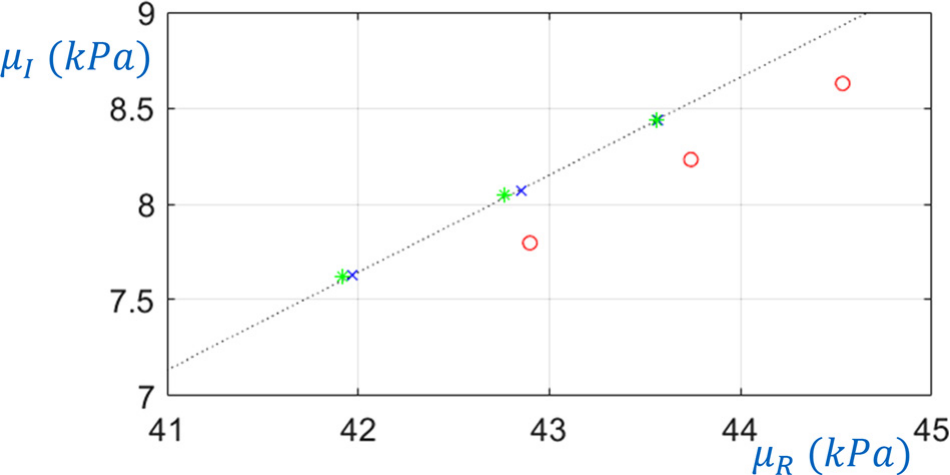
(Color online) Shear loss versus shear storage modulus. The black dashed line is actual value based on Table I values for $\mu_{0},\;\mu_{\alpha },\;\text{and}\;\alpha$ of the Fractional Voigt rheological model. Blue *×*'s are based on the torsionally-polarized curve fit to simulations at 500, 600, and 700 Hz with zero prestrain. Fitted values are also shown for prestrain of 5% without (red circle) and with (green asterisks) the TAE correction.

Curve fits were done in matlab using the “lsqcurvefit” command. Fits for the torsionally-polarized excitation were along 5 cm axial lines, averaging fits from lines spaced 0.5 mm apart from r=0.5 mm to r=R. First, with no prestrain, it was confirmed that this fitting approach conducted at multiple frequencies enabled the identification of $\mu_{0},\;\mu_{\alpha },\;\text{and}\;\alpha$ of the Fractional Voigt rheological model. In Fig. 5, the black dashed line shows the real (storage) part $\mu_{R}$ versus the imaginary (loss) part $\mu_{I}$ of the shear modulus as frequency is varied. The blue *x*'s denote the estimated values of $\mu_{R}$ verus $\mu_{I}$ based on curvefitting FE simulations at 500, 600, and 700 Hz for the 35 mm diameter phantom (they lie on the black dashed line as expected). By drawing a line through these *x*'s, the intercept with the horizontal axis is $\mu_{0}$. The real and imaginary values at a given frequency can then be used to determine $\mu_{\alpha }\;\text{and}\;\alpha$.

For the nonzero prestrain cases, the length distortion was iterated by adjusting $\mu_{\sigma }$ as discussed in Sec. II E until $\xi_{0}^{*}$ at 2.5% prestrain matched $k_{sh}$ (previously obtained with 0% prestrain) minimizing the least square error. It was found that setting, $\mu_{\sigma }=-0.75$, gave the best fit, with a difference in predicted $k_{sh}$ of less than 0.1% as compared to the unstressed case, for both the 35 and 20 mm diameter phantoms, as provided in Tables II and III, respectively. This close agreement was also present for 5% prestrain, but results diverged to about 0.4% and 1.4% difference at 10% and 20% prestrain, respectively, possibly due to geometric nonlinearities that are not accounted for in the linear-based approach. Figure 5 also shows how corresponding estimates of μ change as prestrain is increased to 5% without the TAE correction (red circle) and with the TAE correction (green asterisks).

For the axially-polarized case, first a line fit was done along the radial line within the excitation zone (cuff). It was found that $\mu_{\sigma }=-0.75$ also gave a good fit, with a difference in predicted $k_{sh}$ of less than 0.4% as compared to the unstressed case, for both the 20 and 35 mm diameter phantoms tested at 2.5% and 5% prestrain. This provided confidence in the theoretical developments of Sec. II. Results diverged up to 1.2% difference at 10% and 20% prestrain.

Next, averaged axial line fits were located at 0.5 mm spacing from r=0 to r=R extending 30 to 50 mm distance from the excitation cuff (based on the unstrained geometry) in an attempt to reduce near field effects closer to the source, which may contain larger contributions from *n >* 1 of the $\alpha_{1},\;\alpha_{2},\ldots ,\;\alpha_{\infty }$, $\beta_{1},\;\beta_{2},\ldots ,\;\beta_{\infty }$, and $\xi_{1},\;\xi_{2},\ldots ,\;\xi_{\infty }$ solutions. Then, we averaged fits along radial lines 0<r<R taken at axial distances from 30 to 50 mm from the source in 0.5 mm increments. With $\mu_{\sigma }$ already determined, the length distortion was iterated solely by adjusting $\lambda_{\sigma }$ until at 2.5% prestrain the values of $\beta^{*}$ and $\xi^{*}$ satisfied Eq. (37) where the value used for $k_{sh}$ was based on 0% prestrain. As expected, for this case estimates of $k_{sh}$ under unstressed conditions $\left(\sigma =0\right)$ were not accurate, being 5% too high and 7% too low for the 35 and 20 mm diameter phantoms, respectively. However, by setting $\lambda_{\sigma }=2.0$ it was found that cases with 2.5% prestrain yielded predictions of $k_{sh}$ consistent with the unstressed case within 0.2%. This difference increased to about 6% at 20% prestrain.

For the transversely-polarized (flexural wave) case, fits along axial lines were located in 0.5 mm increments from r=0 to r=R extending 30 to 50 mm distance from the excitation (based on the unstrained geometry), as in the axially-polarized study, in order to estimate $\xi_{1}$. The same length distortion used in the axial study, $\text{Real}\left[\sqrt{1+(3\mu_{0}\varepsilon /\mu [\omega ])(1+3\mu_{\sigma }+\lambda_{\sigma })}\right]$, was applied here in order to estimate $\xi_{1}^{*}$. As in the axially polarized case it is not surprising that the values of $\xi_{1}$ obtained under zero prestress do not align with $k_{sh}$. They are influenced by waveguide effects (small diameter of the phantom relative to shear wavelength). However, as in the axially-polarized case, by setting $\lambda_{\sigma }=2.0$ it was found that cases with 2.5% prestrain yielded predictions of $k_{sh}$ consistent with the unstressed case within 0.4%. This difference increased to only about 1.5% at 20% prestrain. This suggests that the transverse waves are affected by uniaxial prestress in the same way as the axially-polarized waves, and thus may provide a more direct estimate of $\lambda_{\sigma }$.

Theoretical estimates of ξ using “thick” beam theory were within 10% of values found using the finite element analysis (FEA) and reduced in value with respect to increasing prestress, like in the FEA simulations, though to not the same degree observed in the simulations. As expected, thick beam theory was a better match for the 20 mm versus the 35 mm diameter phantom. Thin beam theory results were poor in both cases. This is expected since the wavelengths generated at 600 Hz were comparable to radial dimensions. They would need to be an order of magnitude greater than radial dimensions for thin beam theory to be a good approximation.

To approximate focused modulated radiation force of ultrasound excitation, harmonic 10 mm long line displacements were input at the geometric center of the cylinder in axially- and transversely-polarized directions, as noted in Fig. 1. The response along a line orthogonal to the truncated line source was then fit to determine the complex wavenumber. If we assume that the truncated line source is an infinite plane in an infinite medium (neglecting finite boundaries) and is creating a planar shear wave front the axially-polarized wave traveling in the radial direction with wavenumber β should be governed by

$$\left(1+\frac{\sigma }{\mu }\left(\mu_{\sigma }\right)\right)\beta^{2}=\beta^{*2}=\frac{\omega^{2}\rho }{\mu }=k_{sh}^{2}.$$ (39)

In other words, $\beta^{*}=\beta \sqrt{1+(\sigma /\mu )\left(\mu_{\sigma }\right)}$. This is in agreement with Eq. (28) for the case that ξ=0. Values reported in Tables II and III show that, indeed, $\beta^{*}$ at 2.5% prestrain is consistent with the value of β determined at 0% prestress, but these values differ from $k_{sh}$ by about 5% in terms of the real part, which is inversely proportional to shear wavelength $\left(2\pi /\mathrm{Real}\left[k_{sh}\right]\right)$. The imaginary value for β is an order of magnitude greater than $k_{sh}$. This is expected since, not only is there attenuation due to viscosity, but also due to geometric dispersion as the finite source spreads out in three dimensions, including the axial direction, as it propagates.

Likewise, if we assume that the truncated line source is an infinite plane in an infinite medium (neglecting finite boundaries) and is creating a planar shear wave front the radially-polarized wave traveling in the axial direction (direction of uniaxial prestress) with wavenumber ξ should be governed by

$$\left(1+\frac{\sigma }{\mu }\left(1+\mu_{\sigma }\right)\right)\xi^{2}=\xi^{*2}=\frac{\omega^{2}\rho }{\mu }=k_{sh}^{2}.$$ (40)

In other words $\xi_{a}^{*}=\xi \sqrt{1+(\sigma /\mu )\left(1+\mu_{\sigma }\right)}$. This is not in agreement with Eq. (28) for the case that β=0, where in that case $\xi_{b}^{*}=\xi \sqrt{1+(\sigma /\mu )\left(1+3\mu_{\sigma }+\lambda_{\sigma }\right)}$. Both $\xi_{a}^{*}$ and $\xi_{b}^{*}$ are provided in Tables II and III. We expect the imaginary parts of the wave numbers to be greater than that of $k_{sh}$, which is 54.9 at 600 Hz, since there will be geometric spreading. However, in this case, the geometric spreading is more confined by the small diameter of the phantom relative to its axial length. Imaginary values are larger than $k_{sh}$, but by not nearly as much as they were for the other line segment case. And, as expected since there is less spreading, the imaginary part of the wavenumber is less for the 20 mm versus the 35 mm diameter phantom, since there is less geometric dispersion (∼120 versus ∼180 $m^{-1}$). By looking at the trend of the real part of $\xi_{a}^{*}$ and $\xi_{b}^{*}$ as prestress increases, it appears that $\xi_{b}^{*}$ is the better estimate for $k_{sh}$, suggesting that finite diameter (waveguide effects) have impacted the transverse wave motion.

## IV. EXPERIMENT

### A. Optical elastography methods

**FIG. 6.**
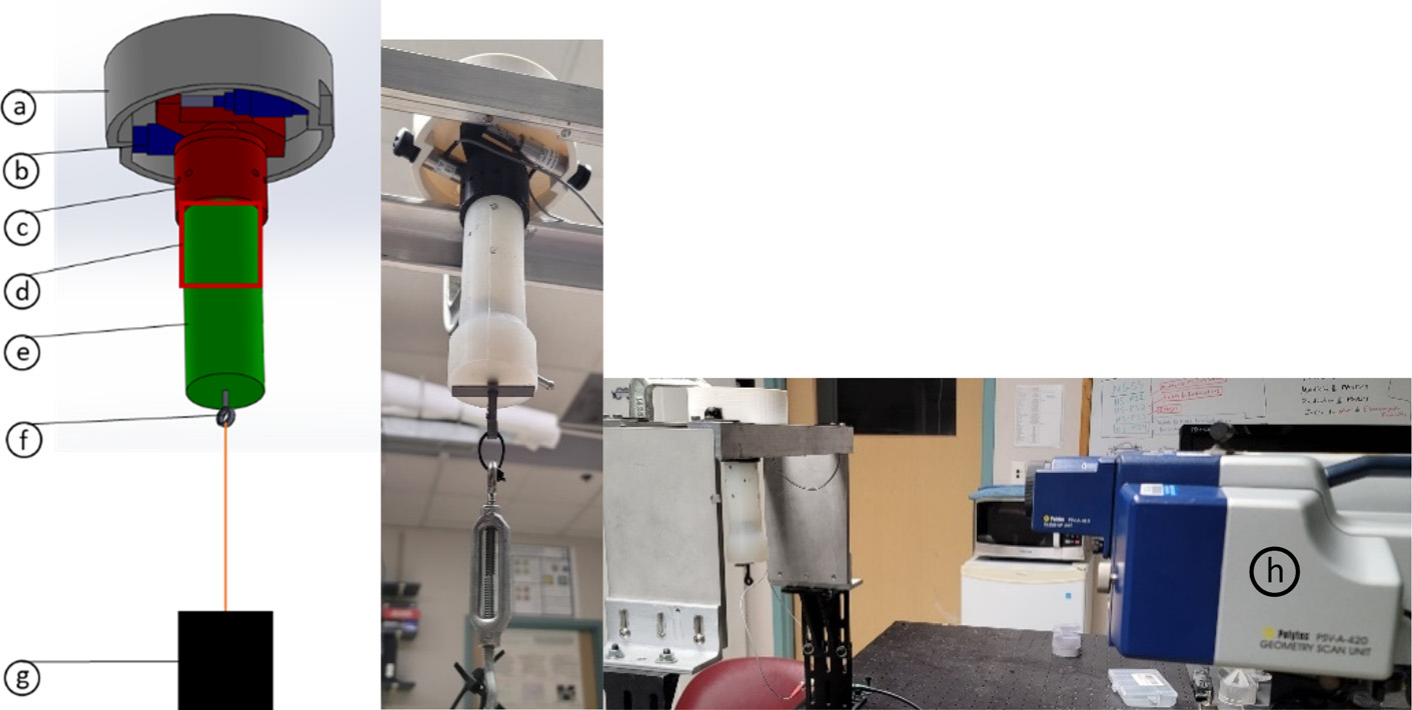
(Color online) Experimental setup for optical elastography using SLDV. Diagram with a bottom view and photo from the bottom and side-angle view showing cylindrical polymer phantom and tensioner. Color-coded view from bottom: (a) Bore constraint cap; (b) piezo stack; (c) rotor hub; (d) image region of interest (ROI); (e) prestressed phantom; (f) tensioner inside phantom; (g) adjustable weight; (h) Polytec SLDV scan head.

The experimental configuration, shown in Fig. 6, enables excitation and measurement of torsionally-polarized wave motion on the surface of the cylindrical phantom that is simultaneously prestrained along its axis. The fixture parts were designed in Solidworks (Solidworks 2021) and three-dimensionally (3D) printed using a fused filament extrusion printer (Prusa Mk3, PRUSA REF, Prague, Czech Republic) on polyethylene terephthalate glycol (PETG). The level of prestrain, introduced by hanging weight (container of water) from the lower end, was measured using a caliper.

A scanning laser Doppler vibrometer (SLDV; PSV-400, Polytec, Waldbronn, Germany), is used to measure motion on the phantom that is in the direction of the laser beam, as described in previous studies.^36,37^ The phantoms used in this study were made of Ecoflex™ 00–30 (Smooth-On, Inc., Macungie, PA) platinum-catalyzed silicone. Ecoflex silicone bases were combined in a 1 A:1B ratio and cured at room temperature in 3D-printed molds in a vacuum chamber (5305-1212, Thermo Scientific-Nalgene, Rochester, NY) to remove any bubbles and improve phantom homogeneity.

Our group has characterized the dynamic shear viscoelastic properties of Ecoflex materials under infinitesimal (0% pre-strain) conditions over a wide frequency range.^35,38^ Previous publications focused on Ecoflex-10^TM^, a similar polymer, with greater viscosity, as compared to Ecoflex-30. For harmonic excitation of both materials using small perturbations about the unstressed state, we've found that a “fractional Voigt” rheological model best describes the frequency-dependent shear storage and loss moduli of the material. The 3-parameter fractional Voigt model is comprised of a purely elastic element of strength $\mu_{0}$ in parallel with a fractional order springpot that is defined by α and $\mu_{\alpha }$ such that, at frequency, $\omega =2\pi f\;\left(\text{rad}/s\right)$, the shear storage $\mu_{R}$ and loss $\mu_{I}$ moduli are given by the following equations:

$$\mu_{R}=\mu_{0}+\mu_{\alpha }\omega^{\alpha }\;\text{cos}\left(\pi \alpha /2\right),$$ (41)

$$\mu_{I}=\mu_{\alpha }\omega^{\alpha }\;\text{sin}\left(\pi \alpha /2\right).$$ (42)

From Ref. 35, it was found that for Ecoflex-10, the following values describe shear viscoelastic properties over the range from 200 Hz to 7.75 kHz: $\mu_{0}=13.3\;\mathrm{kPa}$, $\mu_{\alpha }=2\;\mathrm{kPa}\cdot s^{\alpha }$, and α=1/3. For an Ecoflex-30 sample, measurements conducted in the same way as in the Ref. 24 over the range from 200 Hz to 1 kHz yielded: $\mu_{0}=27\;\mathrm{kPa}$, $\mu_{\alpha }=1.5\;\mathrm{kPa}\cdot s^{\alpha }$, and α=0.3. Thus, while Ecoflex-30 is “stiffer” (higher $\mu_{0}$) under static conditions, due to its lower viscosity the magnitude of its complex shear modulus increases at a slower rate with frequency ( α=0.3), as compared to that of Ecoflex-10 ( α=1/3). Note, while these values for Ecoflex-30 were used in the numerical finite element study of Sec. III, the torsional experimental study described in this section while confirming the appropriateness of the Fractional Voigt model, yielded different parameter values. This could be due to variations in batches of Ecoflex-30, as well as the different experimental configuration.

SLDV measurements were made over a grid in a ROI as indicated in Fig. 6. By driving the two piezoceramic stack actuators (P842.10, PI USA, Auburn, MA) in phase only torsional motion should be excited in the ideal case that the experimental setup is perfectly symmetric in all aspects, which is never the case. It is expected that some flexural (transverse) wave motion will also be excited. If measurements at equal radial distances from the central axis at the same axial (*z*) position, are subtracted from one another (difference) this should double the β=0 torsional mode and eliminate the *n* = 1 flexural mode. If the measurements are added, it should eliminate the torsional mode and double the *n* = 1 flexural mode. This was done before taking line profiles axially to determine $\xi_{0}$(m^−1^) in the torsional case and $\xi_{1}$(m^−1^) in the flexural case from the same measurement.

### B. Optical elastography results

**FIG. 7.**
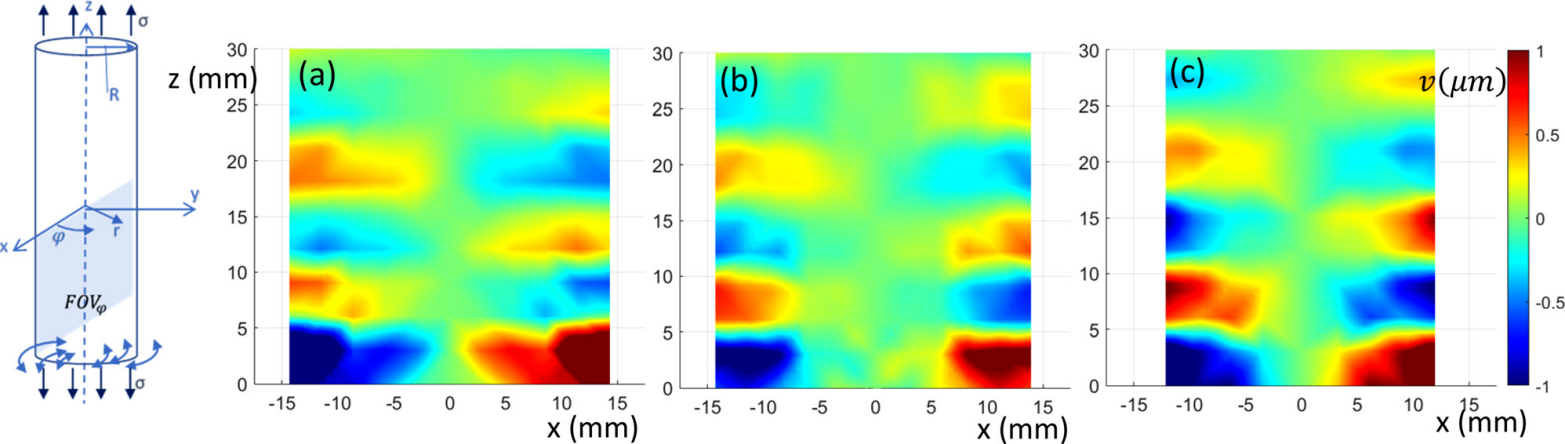
(Color online) Sagittal view of torsionally ( φ)-polarized in-phase wave motion from experimental study. (a)–(c) 0%, 10%, and 20% axial prestrain, respectively. Measurement differs from the FE result in Fig. 2 since the SLDV is measuring motion in the *y*-direction on the rounded surface of the cylindrical phantom, not at a central sagittal cross section.

Optical elastography measurements, processed to amplify torsional motion, as described in Sec. IV A, are shown in Fig. 7 at 0%, 5%, and 10% axial prestrain for the 35 mm diameter phantom. (Image is flipped vertically relative to diagram and photo in Fig. 5.) Corresponding estimates of $\xi_{0}$(m^−1^) in the torsional case are provided in Tables II and III for the 35 and 20 mm diameter phantoms under different prestrain conditions. Estimates of $\xi_{1}$(m^−1^) in the flexural case were found to be unreliable indicating that the torsional motion was significantly greater in amplitude, as was intended. The case with no prestrain yielded an estimate of the shear wavenumber of $k_{sh}=\xi_{0}^{*}=583-j54.4$ (m^−1^) for the 35 mm phantom, and $k_{sh}=\xi_{0}^{*}=568-j110$ (m^−1^) for the 20 mm phantom, suggesting the 35 mm phantom material matched values used in the FEA simulation within 1%, but the 20 mm phantom was slightly stiffer by 3% and significantly more viscous than the material parameter values used in the computational (FEA) simulations, where $k_{sh}=\xi_{0}^{*}=588-j54.9$.

**FIG. 8.**
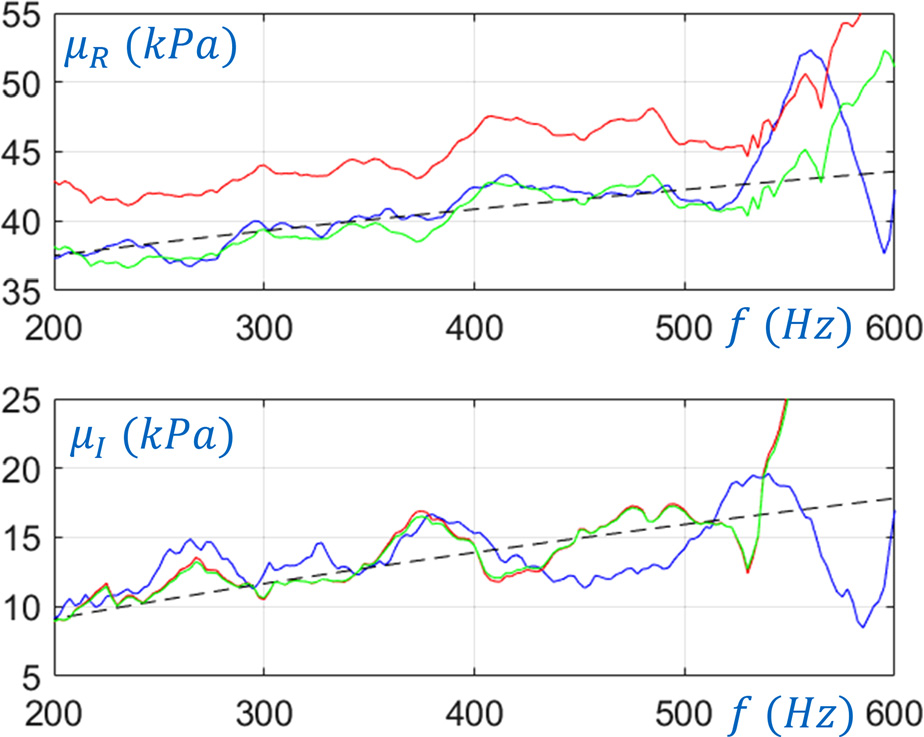
(Color online) The complex shear modulus for the 20 mm phantom based on torsional wave measurements. (a) Shear storage modulus. (b) Shear loss modulus. Key: blue line,  0% prestrain; black dashed line, least square error fit of fractional Voigt model to 0% prestrain case; red line, 10% prestrain; green line, 10% prestrain after length distortion per TAE.

A more comprehensive study of the 20 mm phantom was undertaken by conducting the analysis over the frequency range from 200 to 600 Hz under 0%, 2.5%, 5%, 10%, and 20% prestrain levels in order to fully evaluate the material parameter identification study, starting with identifying the unstressed fractional Voigt model parameters. Figure 8 shows the estimated complex shear modulus $\mu \left[\omega \right]$ under 0% pre-strain (blue lines) based on the experimental estimate of $\xi_{0}\left[\omega \right]=k_{sh}\left[\omega \right]$ and $\mu \left[\omega \right]=\rho {\left(\omega /k_{sh}\left[\omega \right]\right)}^{2}$. The black lines are the corresponding least square error curve fit assuming a fractional Voigt model for $\mu \left[\omega \right]$. The resulting values for the model are $\mu_{0}=31.2\;\mathrm{kPa},\;\mu_{\alpha }=140\;\mathrm{kPa}\cdot s^{\alpha },\;\text{and}\;\alpha =0.613$. As compared to the model used in the numerical studies, this one has similar values for the shear storage modulus, but about twice the shear loss modulus in the frequency range of interest. Here, $\mu \left[\omega /2\pi =600\;Hz\right]=43.6+17.8\;\mathrm{kPa}$. Also, due to unmodeled dynamics in the experimental system, the assumptions necessary for the torsionally-polarized inverse approach start to break down at frequencies above 500 Hz, resulting in large fluctuations in the estimated complex shear modulus.

Figure 8 also shows the experimental estimate of $\mu \left[\omega \right]$ when a 10% prestrain is imposed. As expected, the value of $\mu \left[\omega \right]$ is increased by the prestrain. Distorting the axial length as in the numerical study by dividing it by $\mathrm{Real}\left[\sqrt{1+(3\mu_{0}\epsilon /\mu \left[\omega \right])\left(1+\mu_{\sigma }\right)}\right]$ where $\mu_{\sigma }=-0.5$ results in $\mu^{*}\left[\omega \right]$, denoted by the green line in Fig. 8, which more closely agrees with the value for $\mu \left[\omega \right]$ obtained under the no prestrain case. Table IV summarizes experimentally fit wavenumber estimates $\xi_{0}$ and TAE-adjusted estimates of $k_{sh}=\xi_{0}^{*}$, all based on $\mu_{\sigma }=-0.5$ for the same prestrain levels used in the numerical study.

**TABLE IV.** Wavenumber estimates for 20 mm phantom at indicated prestrain levels.

Prestrain ϵ%	0	2.5	5	10	20
$\xi_{0}$(m^−1^) at **200 Hz**	208-j25.1	199-j23.0	199-j25.5	195-j20.1	195-j24.9
$k_{sh}=\xi_{0}^{*}$ (m^−1^) $\mu_{\sigma }=-0.5$	208-j25.1	202-j23.3	205-j26.3	207-j21.2	217-j27.7
$\xi_{0}$(m^−1^) at **300 Hz**	299-j45.6	292-j45.6	291-j45.7	288-j33.8	279-j36.7
$k_{sh}=\xi_{0}^{*}$ (m^−1^) $\mu_{\sigma }=-0.5$	299-j45.6	296-j46.2	298-j46.9	303-j35.6	308-j40.5
$\xi_{0}$(m^−1^) at **400 Hz**	381-j66.6	375-j74.0	372-j61.1	368-j56.0	349-j46.1
$k_{sh}=\xi_{0}^{*}$ (m^−1^) $\mu_{\sigma }=-0.5$	381-j66.6	380-j74.9	382-j62.6	387-j58.8	383-j50.6
$\xi_{0}$(m^−1^) at **500 Hz**	484-j80.4	469-j79.9	464-j72.4	459-j82.6	419-j116
$k_{sh}=\xi_{0}^{*}$ (m^−1^) $\mu_{\sigma }=-0.5$	484-j80.4	475-j80.1	475-j74.1	480-j86.5	458-j127
$\xi_{0}$(m^−1^) at **600 Hz**	568-j110	513-j189	499-j94.5	476-j113	484-j59.6
$k_{sh}=\xi_{0}^{*}$ (m^−1^) $\mu_{\sigma }=-0.5$	568-j110	519-j191	510-j96.7	497-j118	527-j64.9

For the experimental studies, the value of $\mu_{\sigma }=-0.5$ provided a better fit than the value of $\mu_{\sigma }=-0.75$ used in the FEA studies. The value of $\mu_{\sigma }=-0.5$ is consistent with the initially-developed theory of acoustoelasticity articulated by Biot in his seminal text.^39^ More recent studies have shown that Biot's theory is a special case of a broader theory that also incorporates higher order effects.^23^ Experimental results in Tables II and III show that higher order terms are necessary for accuracy as prestrain increases with the estimate of $k_{sh}=\xi_{0}^{*}$ deviating by more than 6% at 20% prestrain.

### C. Magnetic resonance elastography methods

Axially- and transversely-polarized wave propagation in the same uniaxially prestressed cylindrical isotropic phantoms used in the optical measurements was studied experimentally using magnetic resonance elastography. All experiments were conducted in a 30 cm horizontal bore Agilent 9.4 Tesla preclinical magnetic resonance imaging (MRI) system using a 120 mm I.D. 600 mTesla/meter maximum strength gradient set and a 72 mm I.D. linear radio frequency coil.

**FIG. 9.**
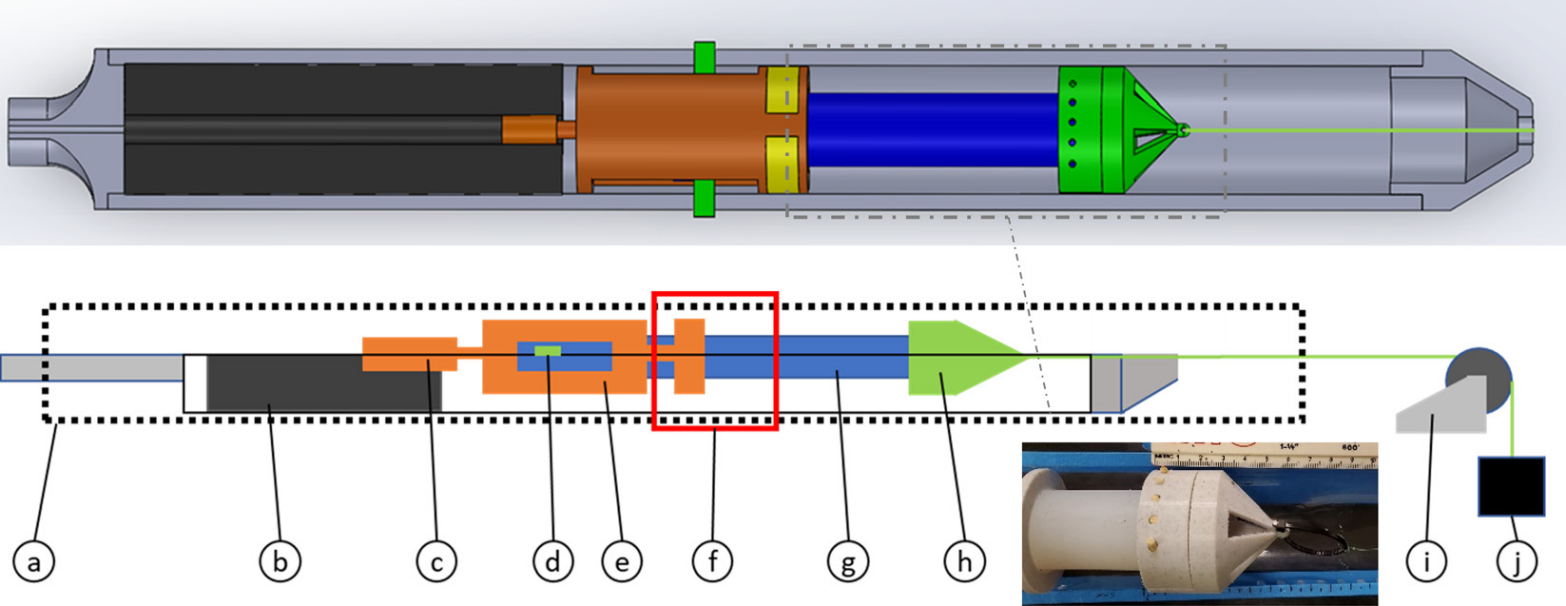
(Color online) Experimental setup for magnetic resonance elastography (MRE). Diagram with top and side view, and photo from top showing cylindrical polymer phantom and claw-grip tensioner. Color-coded view from side: (a) Bore constraint; (b) piezo counter mass; (c) piezo stack; (d) fixed phantom end clamp; (e) harmonic actuator; (f) image ROI; (g) prestressed phantom; (h) claw-grip tensioner; (i) pulley (outside magnet); (j) adjustable weight.

The experimental fixture to hold the phantom in the magnet and simultaneously apply a known static tensile preload while also delivering vibratory excitation via a rigid cuff around the phantom at its mid-length is shown in Fig. 9. The fixture parts were designed in Solidworks (Solidworks 2021) and 3D printed using a fused filament extrusion printer (Prusa Mk3, PRUSA REF) on PETG to limit interference with magnetic resonance (MR) signals and keep parts light enough to be moved efficiently by the piezo stack actuator that delivered the vibratory excitation. It is an advancement of a prior design.^40^ Tensile pre-loading of the cylinder is shown in green in Fig. 9. Samples are gripped by two clamp types: a fixed clamp at the proximal end, and the tensioner clamp at the distal end. Both clamp types are slotted to allow wooden skewers to penetrate the sample, providing reasonably distributed tension, rather than surface tension that would be only available with typical clamps. The distal clamp is then attached to a reinforced nylon wire that is fed out the back of the magnet bore and attached to a pulley system where adjustable weight applies appropriate tension to achieve a desired measurable strain.

Simultaneous harmonic actuation (orange in Fig. 9) is achieved through a piezo stack (P842.10, PI USA LP) located proximally to the entry of the bore to limit interference from wiring, and attached to a Delrin countermass to maximize transference of motion to the phantom. The phantom is placed in a cylindrical tube and the actuator is attached directly to the piezo and slotted for adjustable cuffs (yellow) that uniformly and circumferentially grip the sample, providing a fixed location of the wavesource that contacts the phantom over 15 mm in length axially. Harmonic vibration is produced along the same axis as the pre-tension. Note, however, that because of gravity and imperfections in this setup flexural (transverse) motion will also be excited.

SLIM MRE^41^ was used to acquire vibratory motion encoded in three orthogonal directions simultaneously. Sequence parameters were as follows: TR/TE 1600/16 ms; eight time steps were captured at even intervals over the period of the vibration frequency. A 250 mT/m motion encoding gradient (MEG), which was matched to the vibration frequency, was used with ten cycles. Measurements were repeated with inverted polarity gradients to subtract static field inhomogeneities. The data matrix size of 64 × 64 × 40 with an isotropic voxel size of 0.75 mm resulted in a field of view (FOV) of 48 mm × 48 mm × 30 mm. The piezo stack provided an input axial harmonic motion of ∼10 *μ*m peak amplitude at 600 Hz.

### D. MRE results

**FIG. 10.**
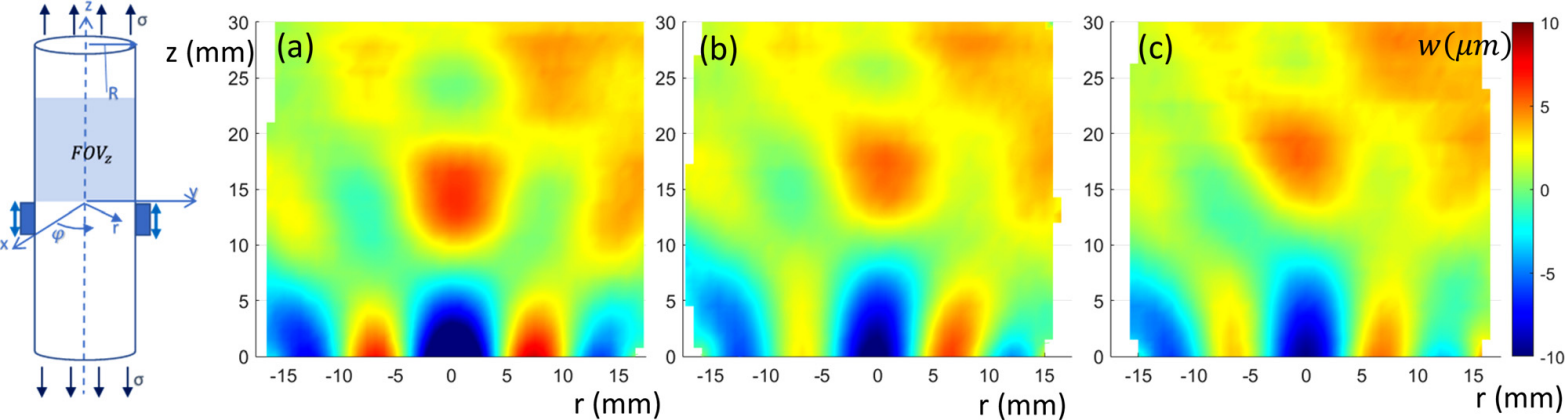
(Color online) Sagittal view of axially (*z*)-polarized in-phase wave motion from experimental study. (a)–(c) 0%, 5%, and 10% axial prestrain, respectively.

Sagittal views obtained using MRE of the axially-polarized vibratory motion are provided in Fig. 10 for 0%, 5%, and 10% axial prestrain for the 35 mm diameter phantom. The field of view is the same as in the numerical study of Sec. III (except shortened to 3 cm along the axis). The images were analyzed using the same TAE approach detailed in Sec. III for the numerical study, with calculation results provided in Tables II and III. Estimates of β based on the radially converging axially-polarized wavefront within the cuff yielded results similar in value to the torsional studies for the 35 mm phantom, given that $k_{sh}=\beta^{*}$. Like in the numerical studies, as prestrain is increased we find that the measured β value increases, as expected per equation (37). A corrected estimate of $\beta^{*}$ can be obtained by dividing the diameter by $\mathrm{Real}\left[\sqrt{1+\mu_{\sigma }(3\mu_{0}\epsilon /\mu \left[\omega \right])}\right]$. “Corrected” values $\beta^{*}$ for nonzero prestrain cases more closely match the zero prestrain case, as can be seen in the last row of Tables II and III. Corrections were made based on the experimentally-estimated rheological model found with the multi-frequency torsional study.

Given the limited FOV in the experimental study, it was found that estimates of ξ or $\xi_{1}$ based on axially- or transversely-polarized (flexural) wave analysis were not reliable. In the finite element study, curve fits for these wavenumbers were based on wave motion 30 to 50 mm from the source, whereas in the experimental study the FOV only extended to 30 mm from the source. Signal-to-noise ratio (SNR) was too poor beyond that range.

## V. DISCUSSION AND CONCLUDING REMARKS

The theoretical, numerical finite element and experimental studies of the previous sections have explored the confounding effects of finite dimensions and nonzero prestress on the elastography approach to estimating material viscoelastic properties in an isotropic cylindrical structure under uniaxial normal stress aligned with the cylinder axis. Additionally, a coordinate transformation approach—TAE—was introduced to estimate material viscoelastic properties independent of the prestress condition without requiring *a priori* knowledge of either the viscoelastic properties or stress conditions. Rather, only the amount of deformation, or strain, from the unstressed condition is required. Once viscoelastic properties are calculated, prestress can also be estimated.

The numerical and experimental studies show both the promise and implementation challenges of elastography and the TAE approach. In the numerical FEA study of Sec. III, the material shear storage $\mu_{R}$ and loss $\mu_{I}$ moduli, as well as the uniaxial normal stress σ could be determined with accuracy within a few percent based on torsional- or axially-polarized excitation, though accuracy generally degrades as the prestrain reaches 10% and 20%. The TAE approach formulated in Sec. II is inherently based on an assumption of linearity and as deformation increases accuracy will suffer. The approach could be improved by accounting for higher order terms in the quasi-static deformation model; this is left for future study. Related to this, in the present study it is assumed that the constitutive relation of elastic materials can be generalized to viscoelastic materials by substituting the real modulus with the complex modulus even if this linearization of the dynamic problem occurs after a finite static deformation (prestrain) has been applied. Further study is needed to evaluate this assumption.

A noted “divergence” between the FEA-based and experimental studies was the observation that the parameter $\mu_{\sigma }$ was best approximated as −3/4 in the FEA simulation, whereas a value of –1/2 did better in the experimental study. Recall that this term accounts for the linear dependence of μ on σ. In the Refs. 22 and 23 it is often denoted $\beta_{1}$ and is related to *A* used in other references,^24–26^ where its value in soft tissue-like nearly incompressible materials can range between negative and positive values. It is hypothesized that its value depends on microstructure and thus can reveal material changes not captured by μ.^24^ Its approximate value of –1/2 in the experimental studies falls within the range found in the literature. The value of −3/4 found in the FEA studies, relative to –1/2, would lessen the effect of the prestress. This is commensurate with the fact that the linear material model used in the numerical study produces less of a change in stress levels for a given strain than does the Gent model, which has been shown to be accurate for the phantom material, Ecoflex-30.^33^

Whereas the unprestressed torsional FEA studies perfectly reproduced the implemented rheological model over multiple frequencies, the multi-frequency experimental study illustrated the approximate nature of the rheological model under no prestress conditions. A least square error fit over the range from 200 to 600 Hz was used to estimate $\mu_{0},\;\mu_{\alpha },\;\text{and}\;\alpha$. This in turn was used in estimating the value of $\mu_{\sigma }$. If the baseline (unprestressed) rheological model is a poor fit, estimates of $\mu_{\sigma }$ based on it would, of course, be inaccurate, as well.

Another source of error is the confounding effect of multiple wave types being present. This is particularly true for the axially-polarized and transverse-(flexural) polarized cases. Selecting a field of view away from the source and filtering can help with some of this, but not all of it. Even in the finite element studies, estimates of material shear properties based on flexural waves at any prestrain level were not accurate.

Simulations of a localized line segment source, an approximation of focused modulated radiation force of ultrasound that is commonly used in ultrasound-based elastography, highlighted how both small dimensions and prestress can alter estimates of the shear viscoelastic properties if those estimates are based on assuming bulk shear wave propagation and do not account for boundary and preloading effects.

Some “next steps” for advancing the strategy introduced here to decouple prestress and waveguide behavior from material shear stiffness estimates include consideration of more complex geometry and stress conditions, as well as anisotropic and nonuniform material properties. Finite element models based on medical images that can provide detailed geometry and localized deformation information under varying loading conditions and, if needed, measures of anisotropy and inhomogeneity, may provide a way to advance the TAE technique beyond simple geometries and assumptions of isotropy and homogeneity.^42^

## Data Availability

The raw numerical and experimental data used in this study is available from the corresponding author upon request.
